# Rapid Diagnosis of Drug Resistance to Fluoroquinolones, Amikacin, Capreomycin, Kanamycin and Ethambutol Using Genotype MTBDRsl Assay: A Meta-Analysis

**DOI:** 10.1371/journal.pone.0055292

**Published:** 2013-02-01

**Authors:** Yan Feng, Sijun Liu, Qungang Wang, Liang Wang, Shaowen Tang, Jianming Wang, Wei Lu

**Affiliations:** 1 Department of Epidemiology and Biostatistics, School of Public Health, Nanjing Medical University, Nanjing, People’s Republic of China; 2 Department of Epidemiology, Kangda College, Nanjing Medical University, Nanjing, People’s Republic of China; 3 Department of Tuberculosis, Zhangjiagang Center for Disease Prevention and Control, Zhangjiagang, People’s Republic of China; 4 Department of Biostatistics and Epidemiology, College of Public Health, East Tennessee State University, Johnson City, Tennessee, United States of America; 5 Department of Chronic Infectious Diseases, Jiangsu Provincial Center for Disease Prevention and Control, Nanjing, People’s Republic of China; St. Petersburg Pasteur Institute, Russian Federation

## Abstract

**Background:**

There are urgent needs for rapid and accurate drug susceptibility testing of *M. tuberculosis.* GenoType MTBDR*sl* is a new molecular kit designed for rapid identification of the resistance to the second-line antituberculosis drugs with a single strip. In recent years, it has been evaluated in many settings, but with varied results. The aim of this meta-analysis was to synthesize the latest data on the diagnostic accuracy of GenoType MTBDR*sl* in detecting drug resistance to fluoroquinolones, amikacin, capreomycin, kanamycin and ethambutol, in comparison with the phenotypic drug susceptibility test.

**Methods:**

This systematic review followed the Preferred Reporting Items for Systematic Reviews and Meta-Analyses (PRISMA) guideline. The search terms of “MTBDRsl” and “tuberculosis” were used on PubMed, EMBASE, and Web of Science. QUADAS-2 was used to assess the quality of included studies. Data were analyzed by Meta-Disc 1.4. We calculated the sensitivity, specificity, positive likelihood ratio (PLR), negative likelihood ratio (NLR), diagnostic odds ratio (DOR) and corresponding 95% confidence interval (CI) for each study. From these calculations, forest plots and summary receiver operating characteristic (SROC) curves were produced.

**Results:**

Patient selection bias as well as flow and timing bias were observed in most studies. The summarized sensitivity (95% CI) was 0.874(0.845–0.899), 0.826(0.777–0.869), 0.820(0.772–0.862), 0.444(0.396–0.492), and 0.679(0.652–0.706) for fluoroquinolones, amikacin, capreomycin, kanamycin, and ethambutol, respectively. The specificity (95% CI) was 0.971(0.961–0.980), 0.995(0.987–0.998), 0.973(0.963–0.981), 0.993(0.985–0.997), and 0.799(0.773–0.823), respectively. The AUC (standard error) were 0.9754(0.0203), 0.9300(0.0598), 0.9885(0.0038), 0.9689(0.0359), and 0.6846(0.0550), respectively.

**Conclusion:**

Genotype MTBDR*sl* showed good accuracy for detecting drug resistance to fluoroquinolones, amikacin and capreomycin, but it may not be an appropriate choice for kanamycin and ethambutol. The lack of data did not allow for proper evaluation of the test on clinical specimens. Further systematic assessment of diagnostic performance should be carried out on direct clinical samples.

## Introduction

Extensive drug resistant tuberculosis (XDR-TB) was first described in March 2006 by World Health Organization (WHO) and Centers for Disease Control and Prevention (CDC) of the United States [1], and has since been reported in more than 50 countries[2]–[4]. WHO has expressed concern over the emergence of XDR-TB and called for measures to prevent the spread of this type of deadly strain [5]. XDR-TB is a rare type of multidrug-resistant TB (MDR-TB) (i.e. resistant to isoniazid and rifampicin) and is resistant to the fluoroquinolones and at least one of three injectable second-line drugs (i.e. amikacin, kanamycin, or capreomycin) [6]. Drug resistance is a severe challenge to tuberculosis control, as it raises the possibility of a condition that can no longer effectively be treated with anti-tuberculosis drugs [7]. Threats of MDR-TB and XDR-TB highlight the urgent need for rapid and accurate drug susceptibility testing (DST) to optimize the treatment regimen and reduce the risk of acquired resistance [8].

Conventional DST for XDR strains is performed sequentially in a two-step procedure beginning with a culture and first-line drug testing, proceeding to further drug testing in the case of multidrug resistance. It takes more than 10 days for traditional culture-based drug resistance detection, even with the new automated liquid media culture systems. For example, the BACTEC MGIT 960 and BACTEC 460TB need 13.3 days and 10.6 days on average to report the drug resistance results, respectively [9]. A rapid, reliable, and accurate test is therefore necessary to avoid clinical deterioration, improve patient management, and prevent further transmissions [10]. During the last decade, a great deal of effort has gone into the development of the molecular-based rapid DST [11], [12]. In 2008, WHO endorsed the line-probe assays (LPAs) for the rapid detection of drug resistance in low and middle income settings [13]. LPAs, in general, focus on detection of drug-resistance gene mutations [14]. The GenoType® MTBDR*plus* and MTBDR*sl* (Hain Lifescience, Nehren, Germany) are two types of LPAs designed for the detection of the first-line and second-line anti-tuberculosis drug resistance, respectively. Both rely on hybridization of amplified DNA fragments from *Mycobacterium tuberculosis (M. tuberculosis)* complex species to specific probes immobilized on nitrocellulose strips. In addition to GenoType® MTBDR*plus* which detects common mutations in *katG gene*, *inhA* promoter, and *rpoB* gene, GenoType MTBDR*sl* detects the most common mutations in *gyrA* gene for fluoroquinolones (FLQs) resistance, in *rrs* gene for amikacin (AM), capreomycin (CAP), and kanamycin (KAN) resistance, and in *embB* gene for ethambutol (EMB) resistance. GenoType® MTBDR*sl* contains 16 probes for mutation detection and 6 probes for quality control. Six control probes consist of a conjugate control (CC), an amplification control (AC), an *M. tuberculosis* complex control (TUB), and three loci controls for gene amplification (*gyrA*, *rrs*, and *embB*) [15]. The remaining 16 probes include wild type gene probes and mutation probes: *gyrA* wild-type probes WT1 to WT3 (codons 85–90, 89–93 and 92–97); *gyrA* mutant probes MUT1, MUT2, MUT3A, MUT3B, MUT3C, and MUT3D for codons A90V, S91P, D94A, D94N/Y, D94G, and D94H, respectively; *rrs* wild-type probes WT1 (codons 1401 and 1402) and WT2 (codon 1484); *rrs* mutant probes MUT1 and MUT2, with A1401G and G1484T changes, respectively; *embB* wild-type probe WT1, covering codon 306; and *embB* probes MUT1A and MUT1B for the mutations of M306I and M306V, respectively [16].

To our knowledge, recent studies have conducted the diagnostic performance of GenoType® MTBDR*sl* in many settings, but the results are inconsistent. The aim of this meta-analysis is to offer a systematic overview on the diagnostic accuracy of GenoType® MTBDR*sl* in detecting drug resistance to FLQs, AM/CAP/KAN and EMB in comparison with phenotypic DST.

## Methods

### Systematic Review

This systematic review was performed according to the guidelines of Preferred Reporting Items for Systematics Reviews and Meta-Analyses (PRISMA) set by the PRISMA Group [17]. This review was registered (registration No: CRD42012002481) in PROSPERO (http://www.crd.york.ac.uk/prospero/), which is an international database of prospectively registered systematic reviews in health and social care.

### Data Resource and Search Strategy

Two investigators independently performed a systematic search based on the PubMed, EMBASE and Web of Science database for original articles published before 1 June 2012. The search items “MTBDRsl” and “tuberculosis” were used. There were no language restrictions. In addition, the bibliographies of each article were reviewed carefully to identify additional relevant articles.

### Inclusion and Exclusion Criteria

Studies that evaluated Genotype® MTBDR*sl* for detection of drug resistance of *M. tuberculosis* to FLQs, AM, CAP, KAN, and EMB were included. Included studies should use the phenotypic DST as a gold standard. The exact number of true-resistance (drug resistance was correctly identified by MTBDR*sl* assay), false-resistance (drug resistance was falsely identified by MTBDR*sl* assay), false-susceptibility (drug susceptive sample was falsely identified by MTBDR*sl* assay), and true-susceptibility (drug susceptive sample was correctly identified by MTBDR*sl* assay) should be available to reconstruct two by two tables. Relevant publications were excluded if they were duplicated articles, reviews (to avoid repeated data), or conference abstracts if the full texts were not available.

### Quality of Studies

The Quality Assessment of Diagnostic Accuracy Studies (QUADAS-2) was used to assess the quality of each study (http://www.bris.ac.uk/quadas/). QUADAS-2 is the current version of QUADAS and the tool for use in systematic reviews to evaluate the risk of bias and applicability of diagnostic accuracy studies. It consists of four key domains: patient selection, index test, reference standard, and flow and timing. Each is assessed in terms of risk of bias and the first three in terms of concerns regarding applicability. Signalling questions are included to assist in judgments about the risk of bias [18]. Risk of bias was judged as “low” if the answers to all signal questions for a domain were “yes”, as “high” if any signal question in a domain was “no”, or as “unclear” if insufficient information was provided [18]. Concern about applicability was assigned as “low”, “high” or “unclear” with the similar criteria.

### Data Extraction

Two investigators reviewed the articles independently. Information was extracted on author, publication year, country (where the specimen came from), specimen type, sample size, gold standard, the number of true-resistance, the number of false-resistance, the number of false-susceptibility, and the number of true-susceptibility to each drug.

### Meta Analysis

We used Meta-Disc 1.4 (http://www.hrc.es/investigacion/metadisc_en.htm) to analyze data [19]. Heterogeneity was identified by using chi-square test and I^2^ (P<0.05 and I^2^>50% indicated significant heterogeneity) [19]–[21]. According to the results of heterogeneity testing, we chose an appropriate statistic model (random or fixed model) to pool the sensitivity, specificity, positive likelihood ratio (PLR), negative likelihood ratio (NLR), and diagnostic odds ratio (DOR). Sensitivity and specificity and corresponding 95% confidence interval (CI) of each study were calculated according to the reconstructed two by two tables. Pooled sensitivity, specificity, PLR, NLR, and DOR were calculated. Additionally, summary receiver operating characteristic (SROC) curves were plotted. The area under the curve (AUC) and Q* index were also counted to evaluate the overall performance of the diagnostic test accuracy [19], [22]. The AUC of an SROC is a measure of the overall performance of a diagnostic test to accurately differentiate those with and those without the condition of interest. Q* index is defined by the point where sensitivity and specificity are equal, which is closest to the ideal top-left corner of the SROC space. Both values range between 0 and 1, with higher values indicating better test performance. Moreover, in consideration of practical application, subgroup analysis was performed by considering specimen types (clinical specimen or clinical isolates) in this study.

## Results

### General Characteristics of Studies

**Figure 1:**
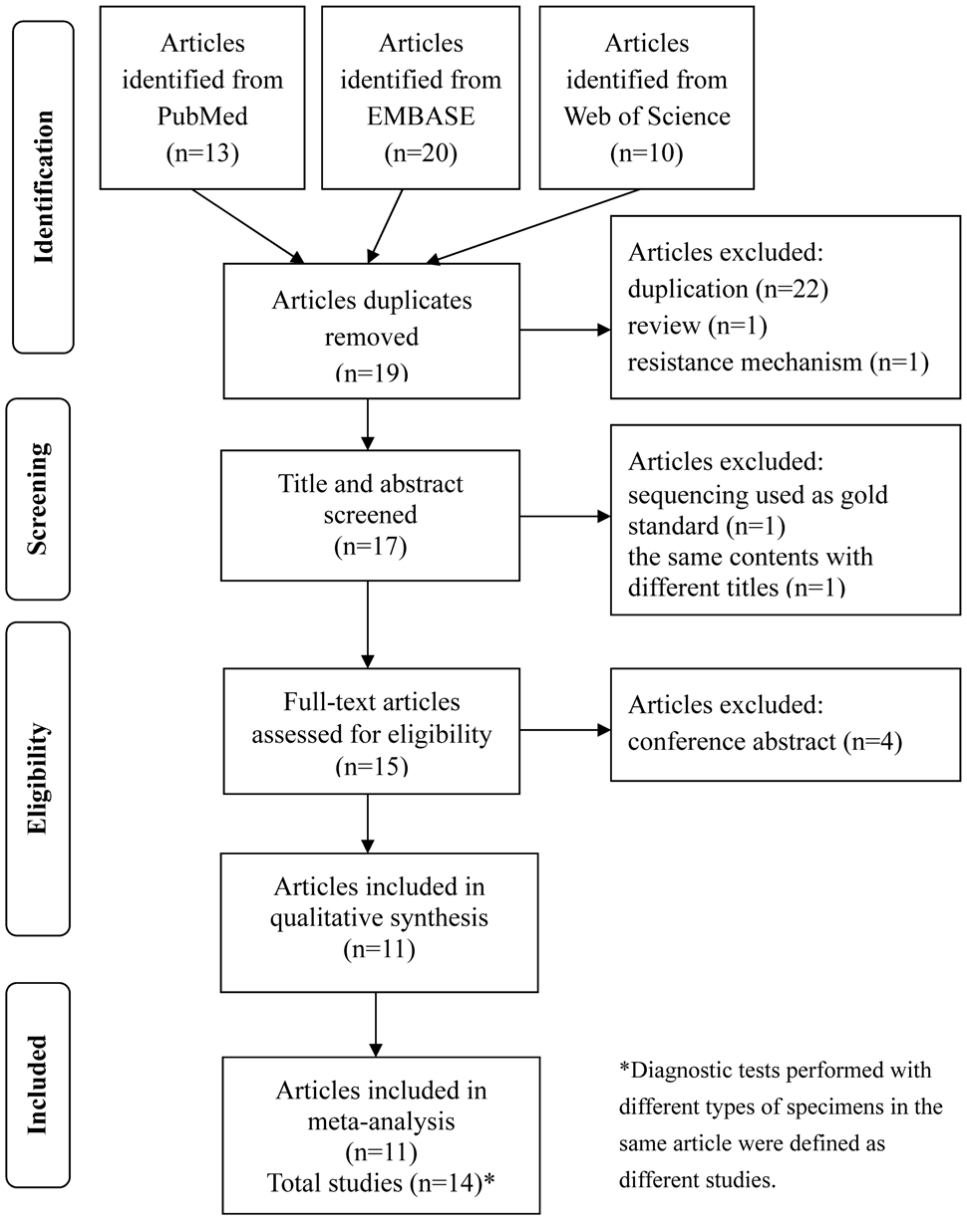
Flow chart of the meta analysis.

A flow chart of inclusion and exclusion procedure of articles is illustrated in Figure 1. In brief, the PubMed search identified 13 articles; the EMBASE search identified 20 articles; and the Web of Science search identified 10 articles. A total of 24 articles was removed due to duplication. Based on the inclusion and exclusion criteria, additional 8 articles were excluded. Finally, 11 eligible articles were included in the meta-analysis and all of them were published in English[15], [16], [23]–[31]. As some articles evaluated more than one Genotype® MTBDR*sl* diagnostic test using different specimen types, we defined 14 independent studies (including 2322 samples) from the 11 articles. Among these 14 studies, 3 studies tested clinical specimens, and others used clinical isolates. Among them, 2 studies were performed in Asia, 2 studies were performed in Africa, 8 studies were performed in Europe, and 2 studies didn’t clearly show the study area. Four types of culture media (L-J PM; agar PM; BACTEC MGIT 960; BACTEC 460TB) were used to perform DST in these studies. We summarized the diagnostic characteristics of these 14 studies in Table 1.

**Table 1:** Summary of included studies.

Study	Country	Specimen type	Size	Gold standard	FLQs				AM				CAP				KAN				EMB				
					TR	FR	FS	TS	TR	FR	FS	TS	TR	FR	FS	TS	TR	FR	FS	TS	TR	FR	FS	TS	
Hillemann 2009 [23]	Germany	Clinical isolates	106	MGIT 960;L-J PM	29	0	3	74	39	0	7	60	39	1	6	60	NA	NA	NA	NA	36	0	16	54	
Hillemann 2009 [23]	Germany	Clinical specimens	64	MGIT 960;L-J PM	8	0	1	51	6	0	2	52	7	0	1	52	NA	NA	NA	NA	10	0	16	34	
Brossier 2010 [24]	France	Clinical isolates	52	L-J PM	21	1	3	27	10	0	0	10	9	1	2	40	13	0	3	39	16	2	12	22	
Kiet 2010 [15]	Viet Nam	Clinical isolates	62	L-J PM	31	0	0	21	NA	NA	NA	NA	NA	NA	NA	NA	5	0	0	57	34	0	19	9	
Van Ingen 2010 [25]	Netherlands	Clinical isolates	29	Agar PM	7	0	0	21	8	0	0	21	7	1	0	21	NA	NA	NA	NA	NA	NA	NA	NA	
Huang 2011 [26]	Taiwan	Clinical isolates	234	Agar PM; MGIT 960	63	0	11	160	16	0	3	215	10	6	4	214	16	0	21	197	91	0	71	72	
Kontsevaya 2011 [27]	Russian	Clinical isolates	51	L-J PM; MGIT 960	25	0	4	19	NA	NA	NA	NA	NA	NA	NA	NA	NA	NA	NA	NA	NA	NA	NA	NA	
Ignatyeva 2012 [28]	Estonia	Clinical isolates	800	MGIT 960	288	21	24	367	147	0	34	526	135	15	14	542	147	0	197	363	479	19	141	142	
Lacoma 2012 [16]	NA	Clinical isolates	34	Bactec 460	4	5	3	17	NA	NA	NA	NA	5	3	0	19	5	3	0	19	14	2	9	9	
Lacoma 2012 [16]	NA	Clinical specimens	54	Bactec 460	3	2	5	42	NA	NA	NA	NA	23	4	0	25	23	4	0	25	22	4	18	6	
Miotto 2012 [29]	Italy	Clinical isolates	175	MGIT 960	42	1	15	116	NA	NA	NA	NA	NA	NA	NA	NA	NA	NA	NA	NA	85	2	37	50	
Miotto 2012 [29]	Italy	Clinical specimens	59	MGIT 960	7	0	0	49	NA	NA	NA	NA	NA	NA	NA	NA	NA	NA	NA	NA	31	0	5	20	
Said 2012 [30]	South Africa	Culture isolates	342	Agar PM	26	7	11	292	NA	NA	NA	NA	NA	NA	NA	NA	NA	NA	NA	NA	27	118	21	150	
Tessema 2012 [31]	Northwest Ethiopia	Clinical isolates	260	BacT/ALERT 3D	NA	NA	NA	NA	NA	NA	NA	NA	NA	NA	NA	NA	NA	NA	NA	NA	8	0	11	241	

Abbreviations: TR = true resistance; FR = false resistance; FS = false susceptibility; TS = true susceptibility; FLQs = fluoroquinolones; AM = amikacin; CAP = capreomycin; KAN = Kanamycin; EMB = ethambutol; NA = not available.

### Quality Assessment

According to QUADAS-2 assess, only three (21%) studies were at low risk of patient selection bias while nine (65%) studies were at high risk of selection bias due to inconsecutive or nonrandom patient selection. The index test bias was minimal compared to patient selection bias. Although four (29%) studies were lacking information to judge, the remaining ten (71%) studies were all at low risk of index test bias. A similar situation was observed in the reference standard bias. Eight (57%) studies were at high risk of flow and timing bias, resulting from the fact that not all selected patients were included in the diagnostic analysis. As for applicability concerns, the overwhelming majority (86%) studies were at high risk of patient selection; however, all selected studies were at low risk of index test and the reference standard. In general, patient selection was the most high-risk bias and high-risk applicability concerns (Table 2).

**Table 2:** Quality assessment of included studies (QUADAS-2).*

Study	Risk of bias				Applicability concerns		
	Patient selection	Index test	Reference standard	Flow and timing	Patient selection	Index test	Reference standard
Hillemann 2009	↑↑	↑	↑	↑↑	↑↑	↑	↑
Hillemann 2009	↑↑	↑	↑	↑	↑↑	↑	↑
Brossier 2010	↑↑	↑	↑	↑	↑↑	↑	↑
Kiet 2010	↑↑	↑	↑	↑	↑↑	↑	↑
Van Ingen 2010	↑↑	↑	↑	↑	↑↑	↑	↑
Huang 2011	↑↑	↑	↑	↑	↑↑	↑	↑
Kontsevaya 2011	↑	↑	↑	↑↑	↑	↑	↑
Ignatyeva 2012	↑↑	↑	↑	↑↑	↑↑	↑	↑
Lacoma 2012	↑↑	↑	↑	↑↑	↑↑	↑	↑
Lacoma 2012	↑↑	↑	↑	↑↑	↑↑	↑	↑
Miotto 2012	?	?	?	↑↑	↑↑	↑	↑
Miotto 2012	?	?	?	↑↑	↑↑	↑	↑
Said 2012	↑	?	?	↑↑	↑	↑	↑
Tessema 2012	↑	?	?	↑	↑↑	↑	↑

* :↑ = low risk; ↑↑ = high risk; ? = unclear.

### Heterogeneity

**Figure 2:**
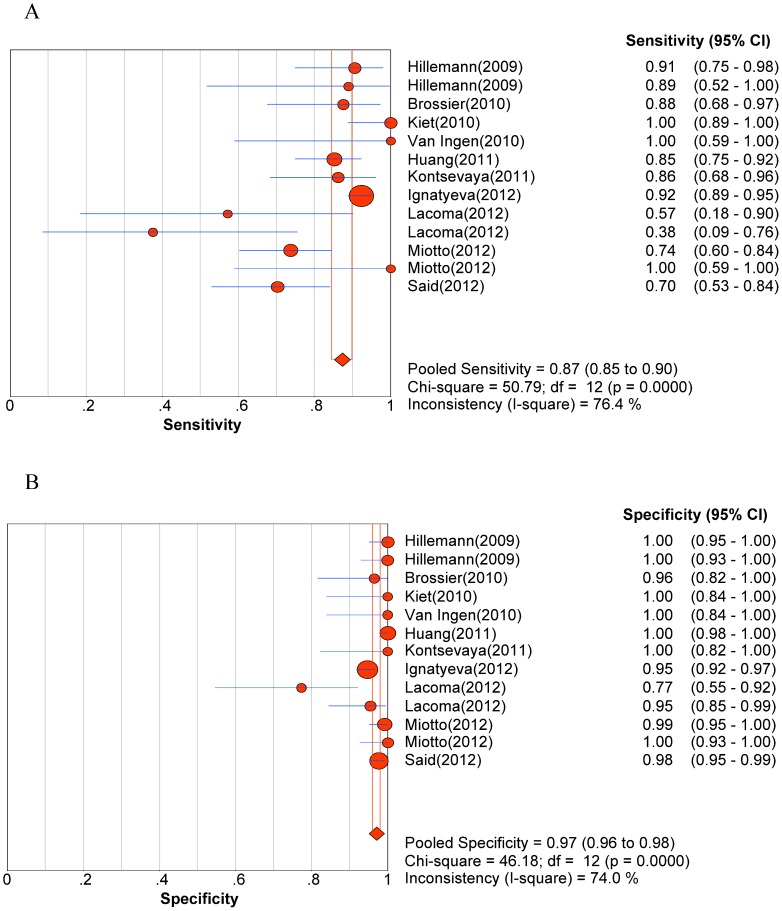
Forest plot of sensitivity for drug resistance to fluoroquinolones. A. Sensitivity; B. Specificity.

**Figure 3:**
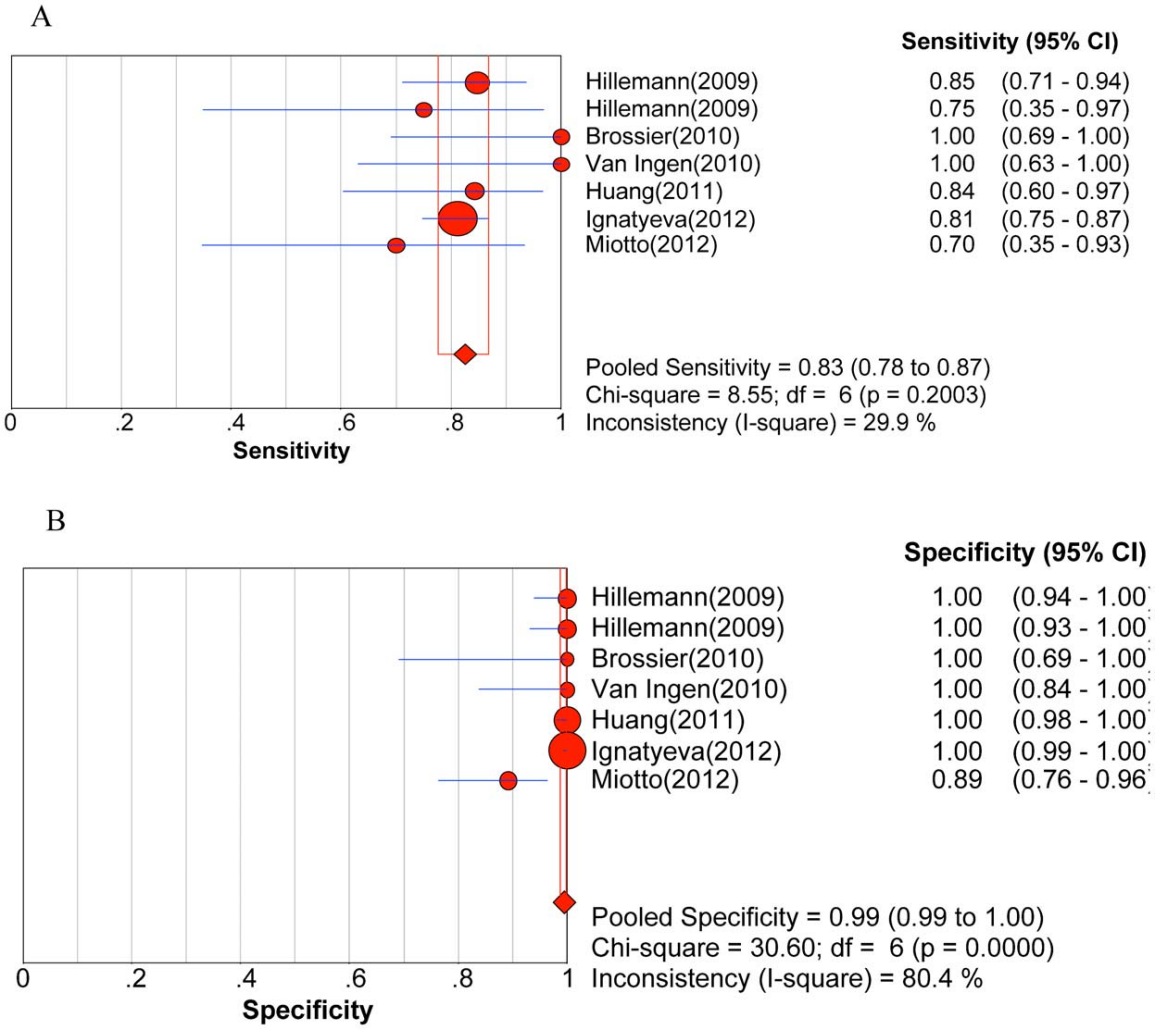
Forest plot of sensitivity for drug resistance to amikacin. A. Sensitivity; B. Specificity.

**Figure 4:**
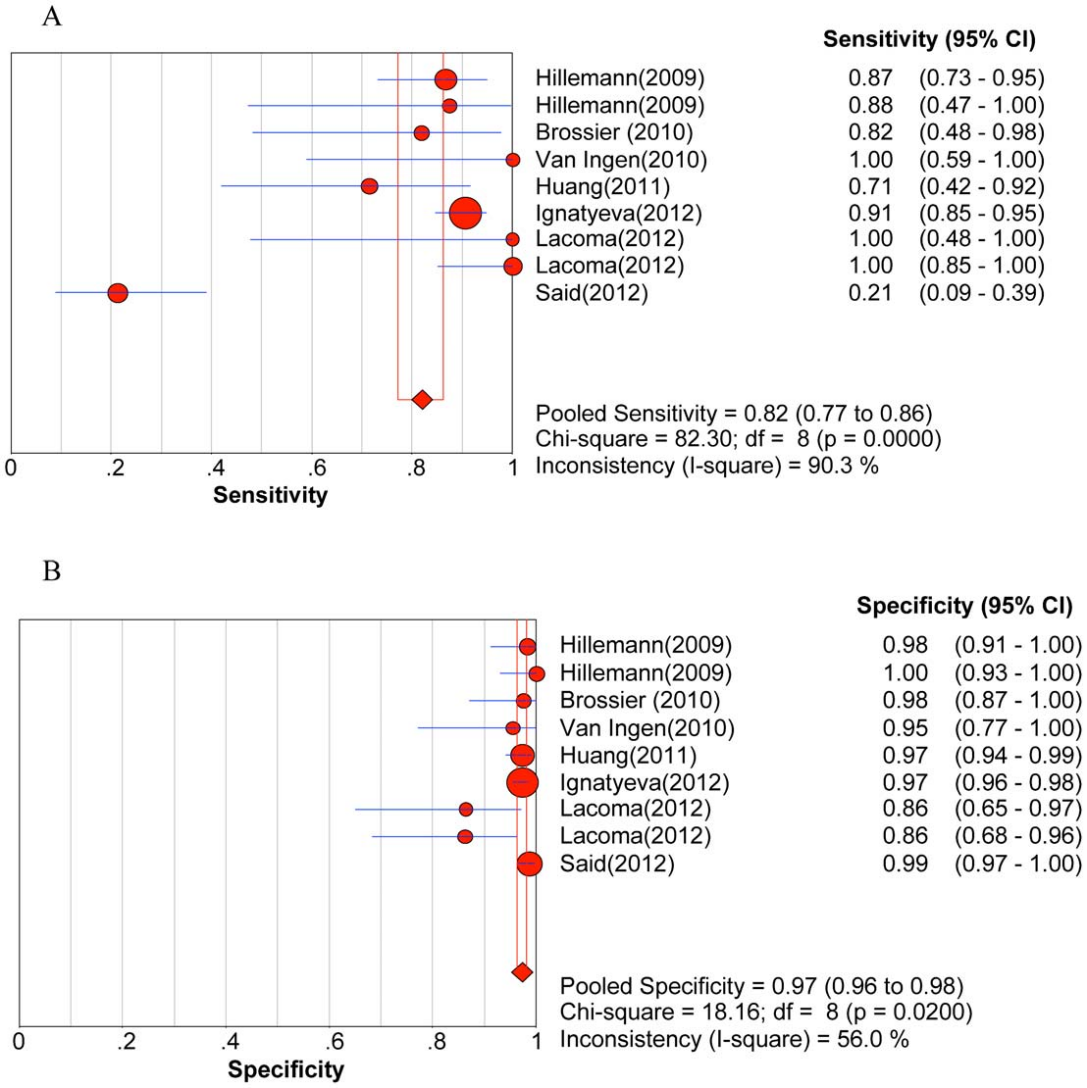
Forest plot of sensitivity for drug resistance to capreomycin. A. Sensitivity; B. Specificity.

**Figure 5:**
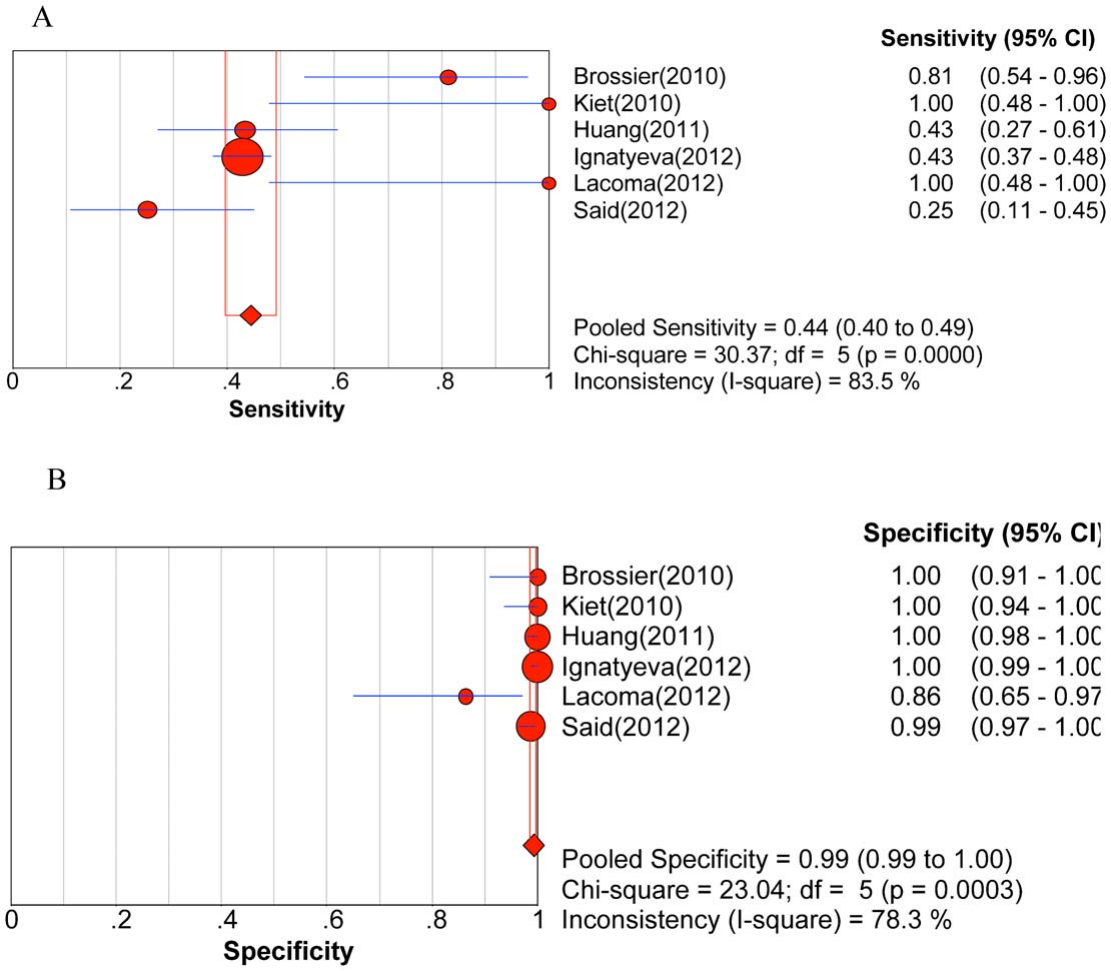
Forest plot of sensitivity for drug resistance to kanamycin. A. Sensitivity; B. Specificity.

**Figure 6:**
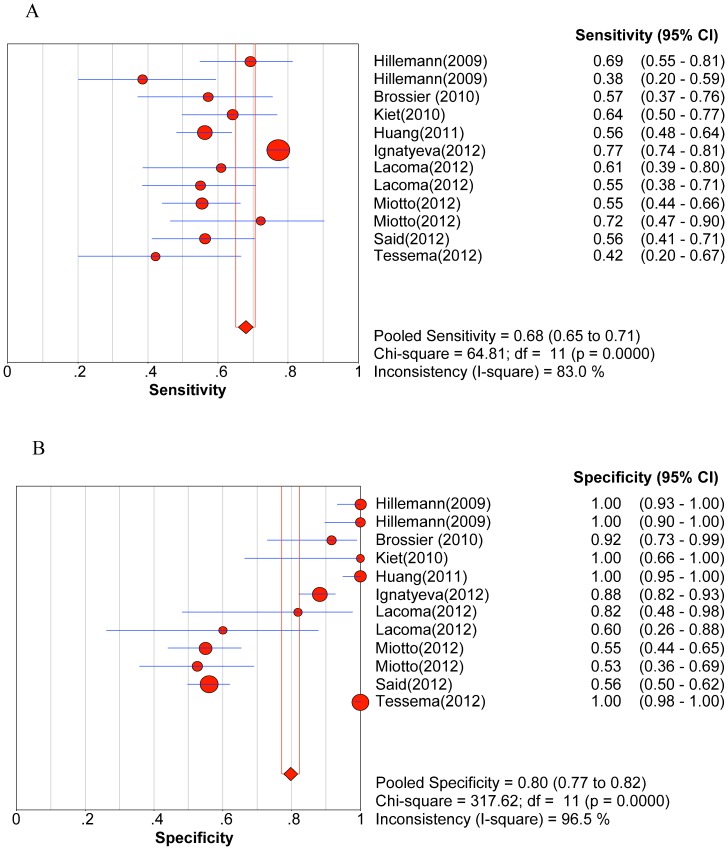
Forest plot of sensitivity for drug resistance to ethambutol. A. Sensitivity; B. Specificity.

Significant heterogeneity was observed when we pooled sensitivity, specificity, PLR, NLR, and DOR of selected studies, except for the sensitivity to AM. The heterogeneity test results of sensitivity and specificity are illustrated in the forest plots (Figure 2, 3, 4, 5, 6).

### Diagnostic Accuracy

**Figure 7:**
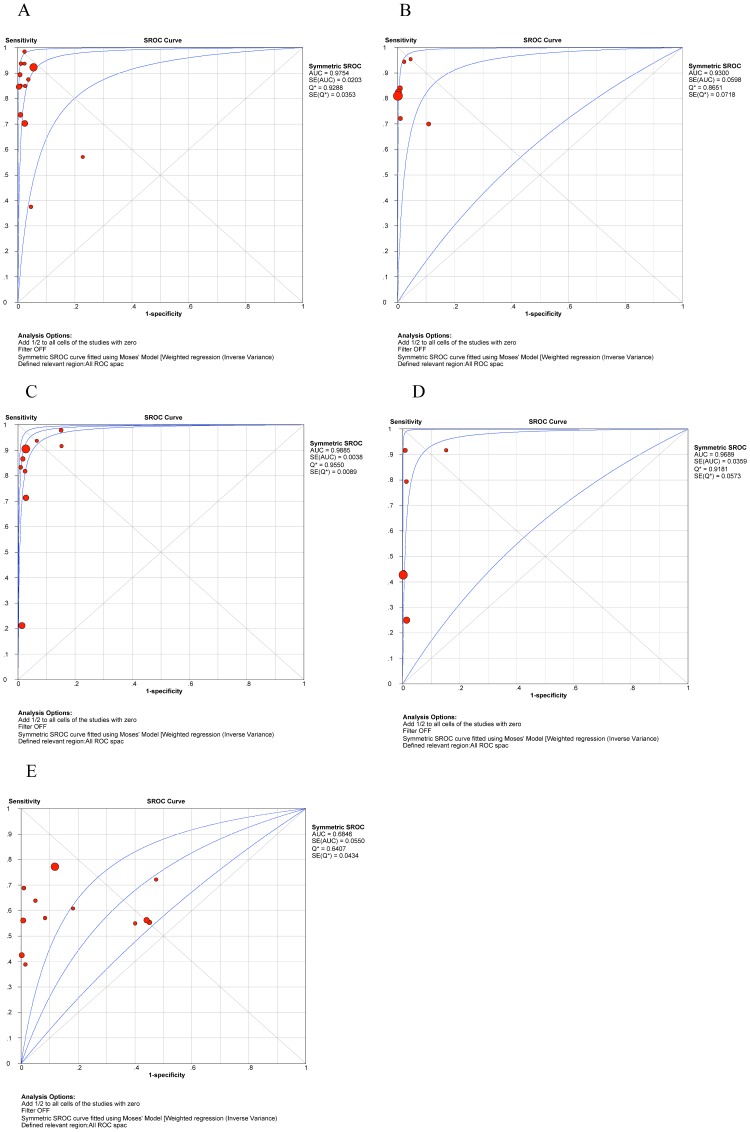
Summary receiver operating characteristic (SROC) curve for drug resistance to fluoroquinolones, amikacin, capreomycin, kanamycin, and ethambutol. A. Summary receiver operating characteristic (SROC) curve for drug resistance to fluoroquinolones B Summary receiver operating characteristic (SROC) curve for drug resistance to amikacin C. Summary receiver operating characteristic (SROC) curve for drug resistance to capreomycin D. Summary receiver operating characteristic (SROC) curve for drug resistance to kanamycin E. Summary receiver operating characteristic (SROC) curve for drug resistance to ethambutol.

The pooled sensitivity, specificity, PLR, NLR, DOR and their 95% CIs are listed in Table 3. The summarized sensitivity (95% CI) of GenoType® MTBDR*sl* was 0.874 (0.845–0.899), 0.826 (0.777–0.869), 0.820 (0.772–0.862), 0.444 (0.396–0.492), and 0.679 (0.652–0.706) for FLQs, AM, CAP, KAN, and EMB, respectively. The specificity (95% CI) was 0.971 (0.961–0.980), 0.995 (0.987–0.998), 0.973 (0.963–0.981), 0.993 (0.985–0.997), and 0.799 (0.773–0.823) for FLQs, AM, CAP, KAN, and EMB, respectively. The AUC (standard error) was 0.9754 (0.0203), 0.9300 (0.0598), 0.9885 (0.0038), 0.9689 (0.0359), and 0.6846 (0.0550) for FLQs, AM, CAP, KAN, and EMB, respectively. Additionally, Q* index (standard error) was 0.9288 (0.0353), 0.8651 (0.0718), 0.9550 (0.0089), 0.9181 (0.0573), and 0.6407 (0.0434) for FLQs, AM, CAP, KAN, and EMB, respectively. The SROC curves (pooled sensitivity against 1-(pooled specificity)) are shown in Figure 7. Figure 2, 3, 4, 5, 6 depicts the forest plots of sensitivity and specificity.

**Table 3:** Summarized diagnostic accuracy of Genotype MTBDR*sl.*

Drug	Se (95% CI)	Sp (95% CI)	PLR (95% CI)	NLR (95% CI)	DOR (95% CI)
FLQs	0.874(0.845–0.899)	0.971(0.961–0.980)	26.368(12.851–54.102)	0.182(0.109–0.303)	176.370(69.230–449.330)
AM	0.826(0.777–0.869)	0.995(0.987–0.998)	68.851(7.845–604.234)	0.192(0.150–0.245)	446.130(66.651–2986.200)
CAP	0.820(0.772–0.862)	0.973(0.963–0.981)	18.211(9.964–33.285)	0.151(0.037–0.609)	143.140(56.896–360.120)
KAN	0.444(0.396–0.492)	0.993(0.985–0.997)	48.693(7.289–325.260)	0.561(0.430–0.732)	163.620(29.811–898.090)
EMB	0.679(0.652–0.706)	0.799(0.773–0.823)	4.879(2.250–10.581)	0.498(0.383–0.648)	12.019(4.189–34.481)

Abbreviations: Se = sensitivity; Sp = specificity; PLR = positive likelihood ratio; NLR = negative likelihood ratio; DOR = diagnostic odds ratio; CI: confidence interval; FLQs = fluoroquinolones; AM = amikacin; CAP = capreomycin; KAN = kanamycin; EMB = ethambutol.

### Subgroup Analysis

According to the type of specimen, 14 studies were classified into two groups for subgroup analysis. Pooled sensitivity, specificity, PLR, NLR and DOR for FLQs, AM, CAP, and EMB are presented in Table 4. As KAN resistance was only performed in the clinical isolates, subgroup analysis was not performed for KAN.

**Table 4:** Subgroup analyses by specimen type.

Drug	Specimen type	Se (95% CI)	Sp (95% CI)	PLR (95% CI)	NLR (95% CI)	DOR (95% CI)
FLQs	Clinical isolates	0.879(0.850–0.904)	0.970(0.958–0.979)	26.399(11.610–60.027)	0.167(0.103–0.269)	192.690(71.070–522.420)
	Clinical specimen	0.750(0.533–0.902)	0.986(0.951–0.998)	31.083(4.631–208.629)	0.225(0.031–1.653)	159.810(6.512–3921.400)
AM	Clinical isolates	0.833(0.783–0.876)	1.000(0.996–1.000)	120.34(28.834–502.202)	0.179(0.138–0.233)	1354.100(321.070–5710.500)
	Clinical specimen	0.722(0.465–0.903)	0.949(0.885–0.983)	16.517(1.211–225.306)	0.310(0.153–0.628)	50.934(4.094–633.740)
CAP	Clinical isolates	0.803(0.750–0.849)	0.975(0.964–0.983)	20.535(11.418–36.933)	0.181(0.038–0.855)	124.310(42.688–361.990)
	Clinical specimen	0.968(0.833–0.999)	0.951(0.878–0.986)	17.159(1.394–211.166)	0.089(0.013–0.589)	361.390(39.769–3284.000)
EMB	Clinical isolates	0.690(0.662–0.718)	0.805(0.778–0.830)	6.919(2.538–18.865)	0.467(0.345–0.632)	17.182(4.720–62.538)
	Clinical specimen	0.536(0.424–0.645)	0.732(0.622–0.824)	1.935(0.743–5.038)	0.631(0.488–0.816)	3.839(0.960–15.355)

Abbreviations: Se = sensitivity; Sp = specificity; PLR = positive likelihood ratio; NLR = negative likelihood ratio; DOR = diagnostic odds ratio; CI: confidence interval; FLQs = fluoroquinolones; AM = amikacin; CAP = capreomycin; EMB = ethambutol.

## Discussion

In this study, we evaluated the diagnostic accuracy of Genotype® MTBDR*sl* in order to identify whether it was a good tool for rapid drug resistance detection. Findings from this meta-analysis indicated that Genotype® MTBDR*sl* had higher values in detecting drug resistance to FLQs, AM, and CAP by considering the diagnostic index.

Drug resistant tuberculosis has been a severe public health issue worldwide. About 440,000 MDR-TB cases and 25,000 XDR-TB cases are estimated to emerge annually, and 150,000 persons with MDR-TB die each year [32]. The 2009 world health assembly resolution has urged WHO member states “to achieve universal access to diagnosis and treatment of MDR-TB and XDR-TB” [32]. Challenges in standardization for conventional DST persist, especially detection time, inoculum size and dispersion of bacillary clumps, subculture bias, testing environment and critical concentration of second-line drug resistance testing [33]. Newer automated liquid media platforms, such as BACTEC system, may be prone to a higher risk of contamination [34]. Molecular DST mostly utilizes Polymerase Chain Reaction (PCR) to amplify mutation-related genes, and it could significantly shorten detection time. The benefits of rapid DST included increased cure rates, decreased mortality, reduced the development of additional drug resistance, and a reduced likelihood of treatment failure and relapse. The emergence of drug resistant tuberculosis has stimulated the development of molecular kits for rapid detection [35]. Since GenoType® MTBC (differentiation of the *M. tuberculosis* complex from cultured material) was available in 2002–2003, GenoType® MTBDR was developed in 2004 and then followed by GenoType® MTBDR*plus* in 2007 and GenoType® MTBDR*sl* in 2009. GenoType® MTBDR*plus* was designed to identify the *M. tuberculosis* complex and its resistance to rifampicin and/or isoniazid from pulmonary clinical specimens or cultivated samples. The identification of rifampicin resistance is enabled by the detection of the most significant mutations of the *rpoB* gene (coding for the β-subunit of the RNA polymerase). For testing the high level isoniazid resistance, the *katG* gene (coding for the catalase peroxidase) is examined. For testing the low level isoniazid resistance, the promoter region of the *inhA* gene (coding for the NADH enoyl ACP reductase) is analyzed. The GenoType® MTBDR*sl* gives the possibility to diagnose patients with MDR-TB to receive information on further antibiotic resistances to fluoroquinolones, aminoglycosides/cyclic peptides and ethambutol. The identification of drug resistance to fluoroquinolones is enabled by the detection of the mutations of the *gyrA* gene. For the detection of resistance to aminoglycosides/cyclic peptides, the 16S rRNA gene (*rrs*) is examined. For the detection of resistance to ethambutol, the *embB* gene (which, together with the genes *embA* and *embC*, codes for arabinosyl transferase) is examined.

In recent years, studies focusing on the diagnostic value of GenoType® MTBDR*sl* were conducted in many settings, but with varied results. Thus, a systematic review is necessary to provide an overall evaluation. Results from this meta-analysis showed that MTBDR*sl* test has a relatively high sensitivity for FLQs, AM and CAP, but not for KAN and EMB. Moreover, high specificity was observed except for EMB, which indicated that EMB susceptible strains or specimens would be identified as resistant ones with a low possibility. Significant heterogeneity was observed when we pooled sensitivity, specificity, PLR, NLR, and DOR of selected studies, except for the sensitivity to AM. Data were pooled by proper models according to the heterogeneity results. To illustrate the overall significance of MTBDR*sl* test, we used multiple index such as AUC, Q* index, and DOR. AUC and Q* index in SROC curve were widely used as the summary index of overall test performance [36]. High AUC and Q* index of FLQs, AM, CAP and KAN except for EMB showed the high accuracy for detecting the resistance to these drugs. The DOR is defined as the ratio of the odds of the test being positive if the subject has a disease relative to the odds of the test being positive if the subject does not have the disease [37]. Higher values of DOR indicate better discriminatory test performance. In this meta-analysis, we observed that DOR of EMB was lower than that of FLQs, AM, CAP and KAN, which indicated that MTBDR*sl* test might not be a good choice for detecting EMB drug resistance. Although SROC curve and DOR could present the overall performance of the test, they are not easy to be used in clinical practice, and the likelihood ratios (LRs) are of more clinical significance [36]. The LRs combine the sensitivity and specificity into a summary index and indicate how much a given diagnostic test result will raise or lower the pretest probability of the target disease [38]. Although in the current analysis, index such as AUC, Q* index, DOR, and PLR showed good performance for KAN resistance detection, its sensitivity was much lower than FLQs, AM and CAP. In other words, more patients with drug resistance to KAN would be misdiagnosed.

Studies have shown that resistance to fluoroquinolones is associated with mutations in a quinolone resistance-determining region of *gyrA* and *gyrB* gene (coding A and B subunits of type II topoisomerase)[39]–[41]. Although Ala-90 and Asp-94 have been the most frequently mutated positions in *gyrA*, Gly-88, Ser-91 and Ala-74 were also reported as the possible mutation sites. However, these potential mutation positions were not all included in the Genotype® MTBDR*sl* strips [42], [43]. Moreover, FLQs stand for a series of antibiotics including ofloxacin, ciprofloxacin, moxifloxacin and gatifloxacin, etc. Moxifloxacin and ofloxacin were the most frequently used drugs in the studies that were involved in this meta-analysis. Mutations in *rrs* gene have been associated with the resistance to AM, CAP and KAN, especially at the positions 1401, 1402 and 1484[44]–[46]. All of these mutation positions were covered by Genotype® MTBDR*sl*. A systematic review has revealed double mutations (for example, A1401G mutation together with A514C, A513C or A1338C) occurred only in resistant strains and has not been reported to occur in any strain susceptible to AM, KAN and/or CAP, whereas the A1401G mutation in *rrs* gene alone was found to occur in up to 7% of CAP-susceptible strains [47]. Cross-resistance between KAN and AM or between KAN and CAP has been observed [45]–[48]. Mutations in *eis* promoter region of *M. tuberculosis* was also reported to be associated with KAN resistance but not being covered by MTBDR*sl* strip [49], [50]. These facts may explain the discordant accuracy results among AM, CAP and KAN although they were tested by one strip with the same mutation positions.

While mutations in codon 306 of *embB* were recognized to be related to EMB resistance [51], [52], the molecular basis of MTBDR*sl* for EMB was not sufficient. Previous studies showed that percentage of *emb*306 mutations in EMB resistant strains varied from 30% to 87.5% [15], [23], [24], [53]. Furthermore, mutations at *emb*306 were reported to be associated with a broad antibiotic resistance rather than EMB resistance [54]. In addition, Huang and colleagues (2012) identified codon 319, codon 497 and other seven novel mutation positions of *embB* gene in the EMB-resistant strains [26]. These facts implied that *emb*306 mutation was not a stable and unique marker for detecting EMB drug resistance. Plinke *et al.* (2009) found that EMB resistant clinical isolates had an increased minimum inhibitory concentration (MIC) as compared to the susceptible ones; but the increase of the MIC was below the value of the critical concentration (2 mg/ml EMB) [55]. Therefore, these strains were regarded as susceptible to EMB by the conventional DST method on Lowenstein Jensen (LJ) media. Previous reports have highlighted the problems of the phenotypic DST on EMB carried out by MGIT [56], [57]. Indeed, EMB testing by MGIT is more affected by lower sensitivity/specificity, lower reproducibility and higher rate of false-positive in detecting resistant cases. In this regard, MGIT as the gold standard when comparing with MTBDR*sl* may under-evaluate the sensitivity and specifity for EMB resistance detection. One paper included in this meta-analysis clearly considered this point providing sensitivity and specificity for EMB resistance adjusted for the results obtained retesting discrepant cases between MGIT and MTBDRsl [29].

There are several limitations of this study. While the bias of patient selection, index test, reference standard and flow and timing were all observed in this meta-analysis according to the QUADAS-2 assessment, most studies (79%) were at high-risk bias in patient selection. The lack of blinding resulted in unclear assessments of bias of index and reference test sections. In addition, regarding data analysis in each study, not all samples included were analyzed because of invalid results, leading to a high risk of flow and timing section bias. As for the review-level, four studies identified by the searching strategy were conference abstract and could not provide exact two by two tables, which affected the pooled data. Moreover, only 3 out of 14 studies tested clinical specimens, providing insufficient data for subgroup analysis for all five drugs.

### Conclusions

Genotype MTBDR*sl* showed good accuracy for detecting drug resistance to FLQs, AM, and CAP of *M. tuberculosis*, but may not be an appropriate choice for KAN and EMB. The lack of data did not allow for proper evaluation of the test on clinical specimens. Further systematic assessment of diagnostic performances should be carried out on direct clinical samples.
